# How to manage residual aortic regurgitation after an aortic valve-sparing operation: Practical tips

**DOI:** 10.1016/j.xjse.2026.100103

**Published:** 2026-02-16

**Authors:** Gift Owolabi, Megan M. Chung, Jack Nickles, William J. MacDonald, Adham Elmously, Hiroo Takayama

**Affiliations:** Division of Cardiac, Thoracic, and Vascular Surgery, Department of Surgery, NewYork-Presbyterian Hospital/Columbia University Irving Medical Center, New York, NY

**Figure:**
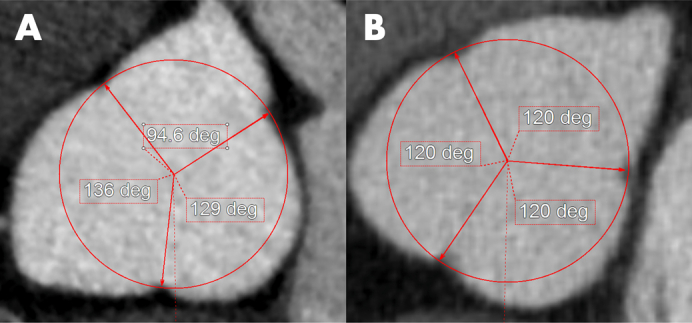
Respecting original cusp orientation facilitates successful valve-sparing operation.

**Central Message**

High-quality aortic valve-sparing surgery requires detailed planning and intraoperative skill. Minimizing residual regurgitation may warrant second aortic crossclamp to achieve satisfactory result.

Valve-sparing root replacement (VSRR) has become a viable procedure for patients with aortic root aneurysm, with excellent outcomes reported in expert hands, particularly in younger and lower-risk patients. As the procedure has matured and excellent long-term data have accumulated in the literature, its unique technical challenges have also been identified. Residual aortic regurgitation (AR) is one such problem, and not to our surprise, the original contribution by Drs David and Feindel^1^ of 10 patients has already provided insight into this topic. They reported no operative deaths, 1 patient with moderate-to-severe AR who ultimately required a Bentall procedure, and 3 patients with postoperative residual mild AR. In a 2017 update of their 20-year experience, residual mild AR was present in more than 20% of patients immediately postoperatively.^2^ Other institutions report similar levels of residual AR^3^^,^^4^; rates of mild AR range between 26% and 60%, with very low rates of moderate or more AR. Residual AR after VSRR may lead to aortic valve reintervention. Rosinski and colleagues^5^ and Kim and colleagues^6^ found residual AR to be associated with progression of regurgitation and aortic valve reoperation, up to 11% at 10 years. These outcomes may be even worse in bicuspid aortic VSRR.^7^ We believe high-quality aortic valve-sparing (AVS) operation requires an occasional second aortic crossclamp to repair residual AR and achieve durable results.

Historically, the practice of AVS surgery followed the development of mitral valve repair, and we continue to learn from the literature on mitral valve repair. Residual mitral regurgitation (MR) after mitral repair is associated with worsening MR and reintervention, and thus second clamp for repair of significant residual MR may be performed.^8^, ^9^, ^10^ Ma and colleagues^11^ reported similar in-hospital outcomes after second crossclamp of any patients with moderate-to-severe residual MR when compared with patients who did not require re-crossclamp. Even more, El-Eshmawi and colleagues^10^ reported their “zero tolerance policy” for post-bypass residual MR, re-crossclamping to repair even mild residual MR when the jet origin is intraleaflet or periprosthetic. Strategies for re-repair were dependent on the cause of residual MR; techniques for resolving suture line-related MR included cleft closure, repair of leaflet perforation, magic stitch, or chordoplasty, whereas more complex techniques including annuloplasty revision and edge-to-edge repair were reserved for patients with refractory regurgitation that did not respond to conventional repair. In expert hands, second crossclamping for residual MR is exercised in approximately 2.8% of mitral repair procedures.^12^

Herein, we share the considerations and modifications we have developed over our experience with residual AR after AVS surgery with reimplantation technique. This manuscript includes a retrospective discussion of institutional patient outcomes. Use of these data was approved by the institutional review board of Columbia University Irving Medical Center (#AAAR2949; approved April 3, 2025), with a waiver of informed written consent.

## Initial Approach

**Figure 1:**
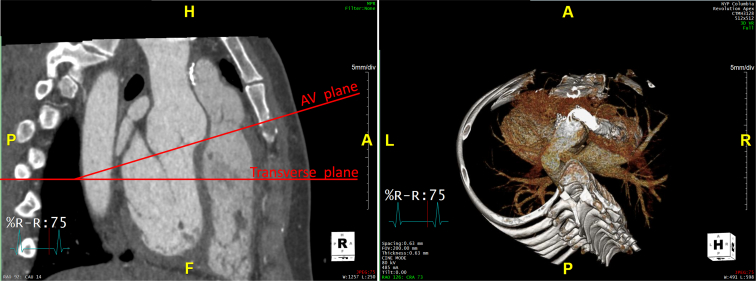
Example demonstrating aortic valve orientation assessment on TeraRecon in lateral (*left*) and superior views (*right*); posteriorly facing valves can be difficult to visualize due to their atypical alignment within the chest.

The best strategy for treating residual AR is to prevent it during initial AVS operation. Because the literature is rich in this topic, we focus on a few unique techniques we use at our Aortic Center. We start with preoperative studying of the aortic valve and root anatomy as well as deliberate planning to address them during surgery. We routinely use electrocardiogram-gated computed tomography angiography (CTA) obtained during diastole for preoperative planning for AVS. Alignment of the plane of the aortic valve as it lies within the chest (eg, which direction the aortic valve is facing) is assessed because, in rare instances, most exaggerated, for example, in patients status post arterial switch operation, the valve may face posteriorly in the chest as opposed to anteriorly toward the surgeon, making aortic valve visualization more difficult or near impossible (Figure 1). Even subtle malalignment could distort cusp relationships and mislead intraoperative aortic valve inspection.

**Figure 2:**
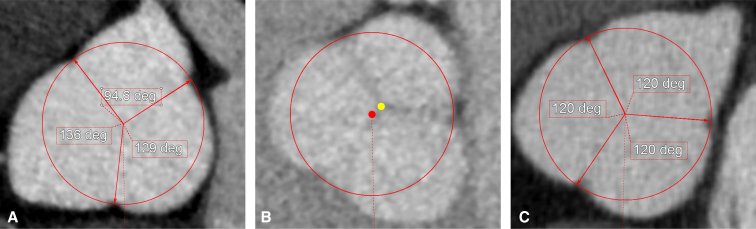
Computed tomography angiography imaging of aortic valves during preoperative assessment. Commissural alignment is often uneven (A). The coaptation point of the 3 cusps (B, *yellow*) may be displaced from the circle connecting the commissures (*red*). We prefer to preserve the native geometry rather than impose a symmetric 120°-120°-120° orientation (C).

In addition, applying principles learned from bicuspid AV repair, we take note of commissural alignment/orientation of tricuspid valves on preoperative CTA. We measure the angulation of the 3 commissural alignments on preoperative CTA (Figure 2, *A*). Our unpublished data show that the alignment is frequently uneven. Of note, the coaptation point of 3 cusps is off center from the circle connecting the 3 commissures (Figure 2, *B*). Because the commissures are reimplanted in a circular graft at VSRR, we measure the commissural alignment angles from the center of circle rather than the coaptation point. We respect the native geometry at the time of commissural reimplantation, rather than forcing all valves into a symmetric 120°-120°-120° configuration, which may lead to frequent need of cusp repair. In a contemporary series at an experienced center, in fact, cusp repair was performed for a quarter of patients undergoing tricuspid aortic valve VSRR with reimplantation.^7^ In our anecdotal experience, respecting the original cusp orientation seems to facilitate tricuspid AVS operation with improved coaptation. In very asymmetrically aligned tricuspid aortic valves, we perform an “isosceles repair” (Figure 2, *C*).

The anatomy and integrity of the cusps are further assessed with transthoracic echocardiography and transesophageal echocardiography (TEE), as previously described^13^; however, they are best assessed under direct vision. In general, unless the cusp is foreshortened due to a pathologic process, we may consider sparing cusps with sufficient tissue quantity in select cases of mildly—and rarely moderately—diseased valves. Frequently, older patients have minor annular calcification or fibrotic thickened cusp edges that we debride in favor of retaining the valve, however, the literature is scarce in supporting such a practice. Intraoperative technical details follow the standard.^14^^,^^15^ Measurement of valve components helps objective assessment as well as subjective decision-making. These components include annular diameter, geometric height, free margin length, and commissural height. Over time, we have gained insight on anatomical variations of these structures, fostering our ability to “eyeball” these components. The effective heights are measured using a dedicated caliper, and cusps are plicated to achieve an effective height of 9 mm or greater.^16^ We prefer central plication for this purpose but also use leaflet resuspension, particularly for patients with leaflet fenestrations (Video 1). With the left ventricular (LV) vent on, normal saline is poured over the aortic valve, and deep coaptation across the coaptation line is visualized through the saline. A drop in the water level indicates cusp malcoaptation, and its mechanism can be visualized through the saline. In rare cases of central AR due to poor coaptation, revision of the root graft to a smaller size may be required (Video 2). Alternatively, when prolonged cardiopulmonary bypass time or a paucity of available healthy tissue is a concern, we frequently use Cabrol annuloplasty. Importantly, excessive LV vent suction can collapse the ventricle and decrease the LV outflow tract and basal ring dimensions; therefore, care should be taken not to misinterpret coaptation under a negatively pressurized state. In addition, when retracting the commissures, attention is paid to not misalign the axis of the root, which may distort the apparent coaptation geometry.

## Assessment of Residual Regurgitation

The first assessment of the aortic valve after AVS operation is the manual LV assessment immediately after aortic declamping. When residual AR is significant, the LV may quickly distend, which requires prompt assessment and reclamping. Once an organized rhythm is restored, the arterial pressure tracing can inform the degree of ejection from the LV, which receives minimum preload on bypass; thus, A-line pulsatility can imply residual AR. Zero or minimum pulsatility is expected with successful AVS operation. TEE assessment of the aortic valve follows. Of note, AR is exacerbated on TEE until the patient is weaned off bypass and normal hemodynamics and blood flow through the aortic valve are restored. Residual AR should be assessed through multiple TEE views, including deep transgastric long-axis view, in which AR often appears worse than in comparison to the midesophageal view. Standard AR quantitative assessment is performed in addition to qualitative assessment of the color Doppler image; however, combinations of different AR mechanisms, such as central and commissural regurgitation, while challenging for quantification, are not uncommon after a repair attempt.

In our algorithm, second aortic crossclamp is entertained only if the patient can safely tolerate it. Moderate or more residual AR calls for aortic re-crossclamping for revision or conversion to aortic valve replacement (AVR). The decision to re-crossclamp for mild AR is contextual. Stephens and colleagues^17^ found that mild AR is generally stable on long-term follow-up, with 85% of patients continuing to have mild AR at a median of 57 months. In our experience, among patients who left the operating room with mild or less residual AR, 77.5% remained stable at 5 years, whereas 22.5% progressed to moderate or severe AR. When examining the role of eccentricity in AR progression, Kim and colleagues^6^ found a 5-year cumulative rate of AR progression and need for AVR to be 64.5% and 14.4% for minimal eccentric residual AR, compared with 13.0% and 1.66% for minimal central residual AR, respectively. Another study, from authors in Belgium,^18^ similarly demonstrated that an eccentric character of the AR jet increased the likelihood of severe recurrent AR during follow-up. This suggests that although central mild AR may be left alone, eccentric mild AR should have a lower threshold for being re-explored, particularly if the initial repair procedure suggests room for re-repair; if requiring multiple complex attempts, its durability should be questioned and AVR should be strongly considered.

## What to do After Second Clamp

The underlying pathology of residual AR revealed with TEE and the assessment of the aortic valve during the initial AVS guide the management strategy after second clamp. If the myocardial function is borderline or the initial repair attempt has exhausted technical options, AVR is chosen. Bentall procedure with explant of the root graft is preferred over AVR since it allows implantation of a larger aortic valve prosthesis. For re-repair, unless residual AR causes LV distension, meticulous attention should be practiced during TEE assessment, which also allows the heart to be perfused before second myocardial ischemia. Typically, a re-repairable valve has remaining cusp prolapse or commissural AR, leading to less-than-moderate AR. Myocardial arrest is reintroduced, maximizing the myocardial protection with a combined use of antegrade, direct coronary ostial, or retrograde delivery as appropriate. The graft is then transected and the valve is examined. Frequently, the main pathology is obvious upon this inspection, when the aortic cusps are at their diastolic positioning. For cusp prolapse, additional central plication works if the prolapsing cusp has excess free margin length. Choosing a different site of the cusp edge may be needed if the tissue is too thin around the already-plicated node of the Arantius. When one cusp edge is riding too high in reference to the other cusp edges of appropriate effective height, it could be lowered by plicating the graft at the sinotubular junction of the corresponding sinus with a 4-0 PROLENE mattress suture.

AR at the commissures can be addressed by narrowing the interleaflet triangle with sutures. Our preferred technique is to place a 4-0 PROLENE mattress suture through the aortic annulus near the base of the interleaflet triangle, which is then passed through the graft close to the commissural line. Another 4-0 PROLENE suture is placed from the annulus of the adjacent sinus to the same commissural line. Tying these sutures will narrow the interleaflet triangle and deepen the commissural coaptation as well as plicate the graft (Video 3). We prefer this maneuver over the classical Cabrol stitch in situations for which aggressive graft plication is required. Commissural AR can also be a result of redundant free margin, in which case shortening the free margin with central plication works well for repair without increasing its effective height.

The outcomes of these patients who were re-crossclamped have yet to be fully elucidated in the literature. In our series, among 17 patients who underwent VSRR and required intraoperative second aortic crossclamp, 6 were ultimately converted to a Bentall, whereas 11 underwent successful re-repair.^19^ Of the re-repair cohort, TEE findings before second crossclamp demonstrated residual moderate-to-severe AR in 6, eccentric mild AR in 4, and mild central AR in 1. In the converted cohort, there was moderate-to-severe AR in all 6. Repair techniques included cusp plication alone in 5 patients, commissuroplasty alone in 1, a combination of both in 4, and release of a previous plication suture in 1. All re-repaired patients had mild or less AR on TEE before leaving the operating room and on predischarge transthoracic echocardiography. On average, patients who underwent VSRR and required re-crossclamping had an aortic crossclamp time of 177 minutes—approximately 35 minutes longer than patients without re-crossclamping. Despite this increased operative complexity, short-term outcomes in the re-crossclamped cohort appear comparable with those in patients not requiring second aortic crossclamp. Among patients with available long-term echocardiogram follow-up, average AR on long-term echo follow-up was trace in re-repaired patients (n = 2) at a median of 56 (range 28-72) months. However, re-crossclamping is not without risk and may increase the likelihood of early complications, including reoperation.^5^ This underscores a clinical trade-off: although a second crossclamp introduces additional operative risk, leaving residual AR at the conclusion of the index repair may predispose to early valve failure.^4^^,^^18^ Further study is needed to define the role of intraoperative re-repair in optimizing durability and preventing repair failure, particularly in comparison with conversion to AVR.

Observational studies have noted a 10-year freedom from valve-related reintervention between 88% and 93% after VSRR.^20^^,^^21^ In a 2-center study from our institution and Emory University, of 781 patients who underwent VSRR, we saw a 12.5% all-cause reintervention rate and 7.0% aortic valve reintervention at 10 years.^22^ Indications for AV reintervention on the native valve included severe AR (52.5%), severe aortic stenosis (12.1%), endocarditis (22.5%), and graft infection (5.0%). In patients who may not be strong candidates for reoperative cardiac surgery, transcatheter aortic valve replacement within the VSRR graft has been described.^23^^,^^24^ This modality will be increasingly used with advances in the technology, such as the Jena valve.

## Conclusions

Overall, we recommend the following to prevent residual AR: understand the anatomy of the aortic valve in detail and the mechanism of preexisting AR. Follow the principles of the David procedure to optimize cusp coaptation. We prefer to respect the patient's native commissural orientation. For moderate or more residual AR or any eccentric AR after repair, scrutinize post-bypass TEE to understand the mechanism of failure; second crossclamp and re-repair if the patient can tolerate it, and convert to Bentall if not. In our experience, second aortic crossclamp for re-repair can be well tolerated in appropriately selected cases; as such, developing an objective algorithm for this decision can help guide a successful AVS practice.

## Conflict of Interest Statement

H.T. reported consultant for Artivion and Edwards. All other authors reported no conflicts of interest.

The *Journal* policy requires editors and reviewers to disclose conflicts of interest and to decline handling or reviewing manuscripts for which they may have a conflict of interest. The editors and reviewers of this article have no conflicts of interest.

## Supplementary Data

Video 1 AV repair adjunct techniques: leaflet resuspension. Resuspension using GORE-TEX suture (CV-7) was used to reestablish a competent coaptation line in a valve with fenestrations. Footage shown occurs during first crossclamp. Video available at: https://www.jtcvs.org/article/S2950-6050(26)00007-0/fulltext.

Video 2 AV repair adjunct techniques: annular stabilization and exchange to smaller graft. Intraoperative reevaluation during VSRR showed persistent aortic regurgitation. The aortic root graft was downsized in order to stabilize the aortic annulus and manage the regurgitation. Footage shown occurs during first crossclamp. Video available at: https://www.jtcvs.org/article/S2950-6050(26)00007-0/fulltext.

Video 3 AV repair adjunct techniques: commissural management – narrowing inter-triangle with commissuroplasty. This patient had persistent AR during VSRR requiring management through central plication of 2 leaflets and a commissuroplasty that narrowed the interleaflet triangle. An appropriate dilator may be used to avoid iatrogenic AS. Footage shown occurs during first crossclamp. Video available at: https://www.jtcvs.org/article/S2950-6050(26)00007-0/fulltext.
