# Supplementary figures and images for: How to manage residual aortic regurgitation after an aortic valve-sparing operation: Practical tips
Source: JTCVS Struct Endovasc. 2026 Feb 16;10:100103. doi: 10.1016/j.xjse.2026.100103 (PMC13244682; doi:10.1016/j.xjse.2026.100103)

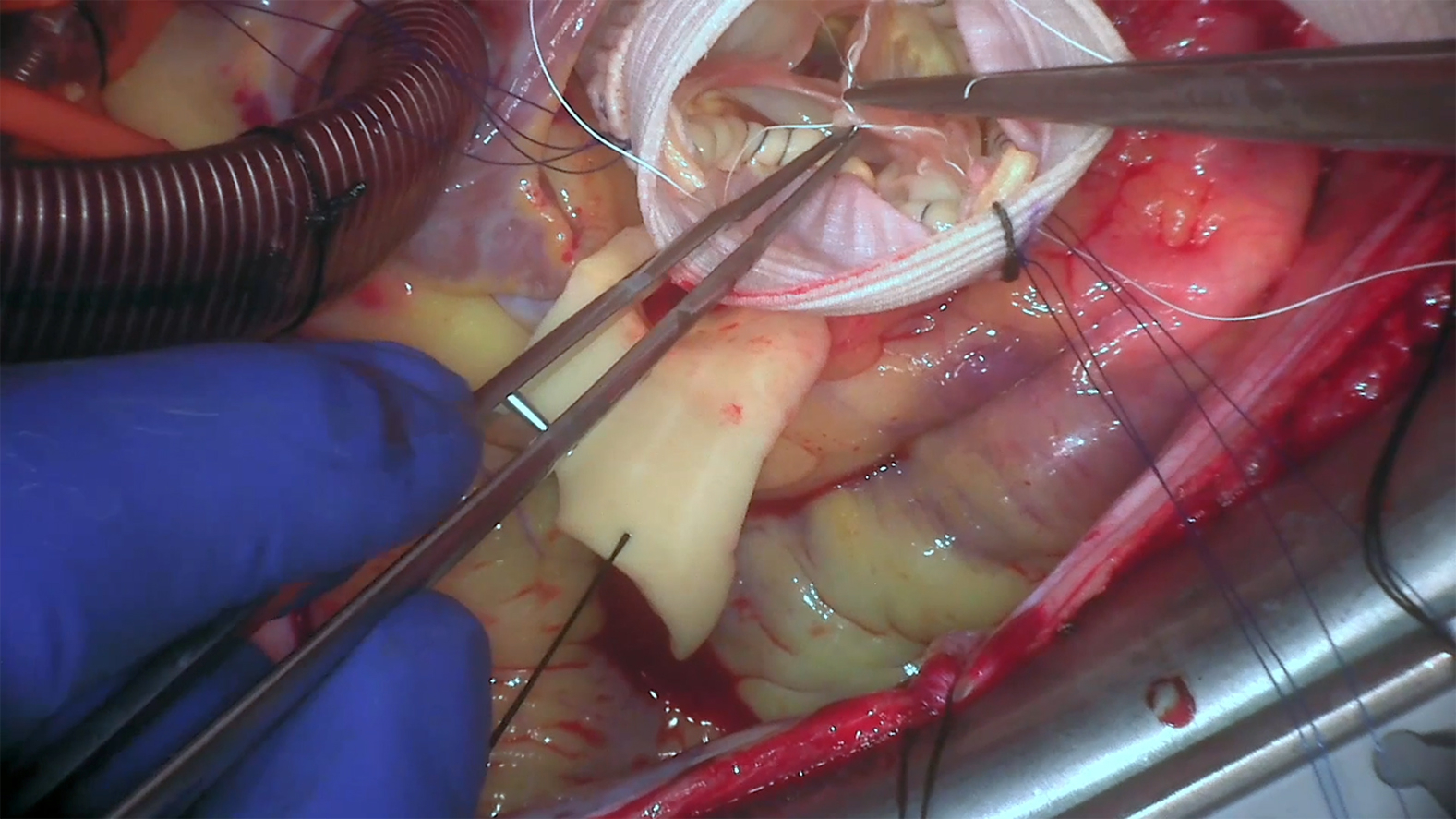

Supplement: Video 1 — AV repair adjunct techniques: leaflet resuspension. Resuspension using GORE-TEX suture (CV-7) was used to reestablish a competent coaptation line in a valve with fenestrations. Footage shown occurs during first crossclamp. Video available at: https://www.jtcvs.org/article/S2950-6050(26)00007-0/fulltext. [file fx2.jpg]

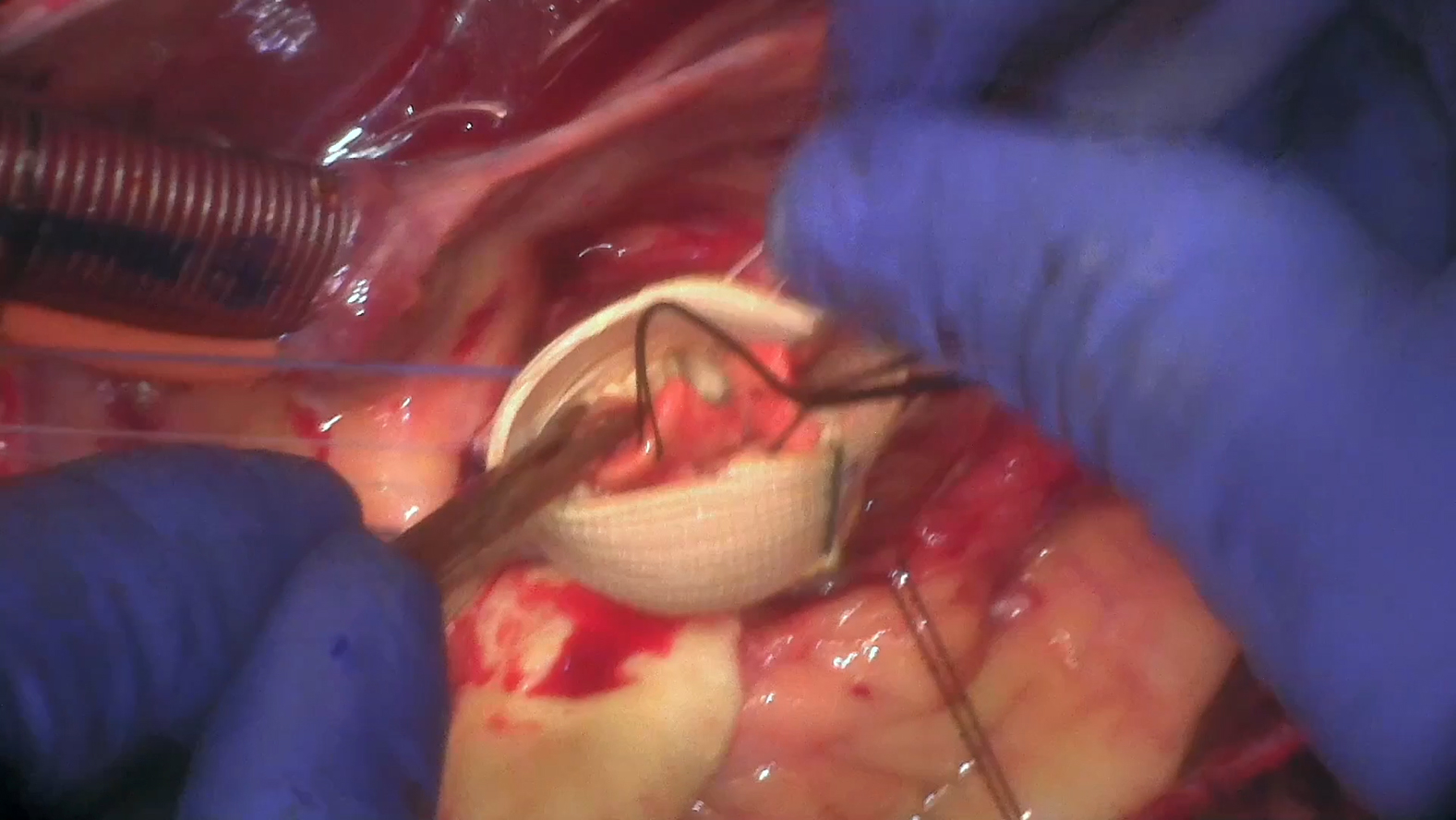

Supplement: Video 2 — AV repair adjunct techniques: annular stabilization and exchange to smaller graft. Intraoperative reevaluation during VSRR showed persistent aortic regurgitation. The aortic root graft was downsized in order to stabilize the aortic annulus and manage the regurgitation. Footage shown occurs during first crossclamp. Video available at: https://www.jtcvs.org/article/S2950-6050(26)00007-0/fulltext. [file fx3.jpg]

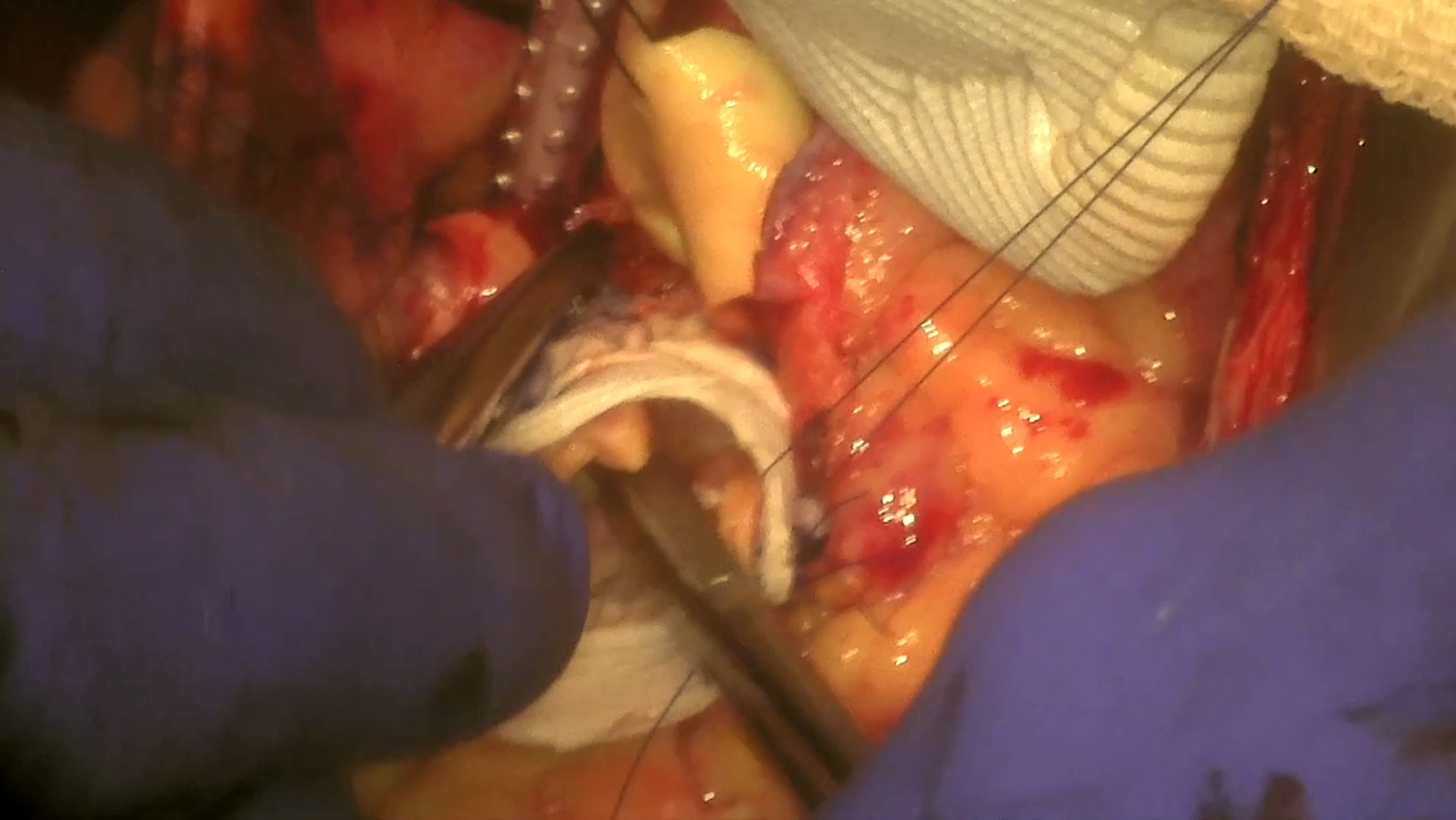

Supplement: Video 3 — AV repair adjunct techniques: commissural management – narrowing inter-triangle with commissuroplasty. This patient had persistent AR during VSRR requiring management through central plication of 2 leaflets and a commissuroplasty that narrowed the interleaflet triangle. An appropriate dilator may be used to avoid iatrogenic AS. Footage shown occurs during first crossclamp. Video available at: https://www.jtcvs.org/article/S2950-6050(26)00007-0/fulltext. [file fx4.jpg]
